# Metabolism of hepatic stellate cells in chronic liver diseases: emerging molecular and therapeutic interventions

**DOI:** 10.7150/thno.106597

**Published:** 2025-01-02

**Authors:** Mengyao Yan, Yimin Cui, Qian Xiang

**Affiliations:** 1 Institute of Clinical Pharmacology, Peking University First Hospital, Beijing, China.; 2 Department of Pharmacy Administration and Clinical Pharmacy, School of Pharmaceutical Sciences, Peking University Health Science Center, Beijing, China.

**Keywords:** hepatic stellate cells, cellular crosstalk, liver diseases, clinical treatment

## Abstract

Chronic liver diseases, primarily metabolic dysfunction-associated steatotic liver disease (MASLD), metabolic and metabolic dysfunction-associated alcoholic liver disease (MetALD), and viral hepatitis, can lead to liver fibrosis, cirrhosis, and cancer. Hepatic stellate cell (HSC) activation plays a central role in the development of myofibroblasts and fibrogenesis in chronic liver diseases. However, HSC activation is influenced by the complex microenvironments within the liver, which are largely shaped by the interactions between HSCs and various other cell types. Changes in HSC phenotypes and metabolic mechanisms involve glucose, lipid, and cholesterol metabolism, oxidative stress, activation of the unfolded protein response (UPR), autophagy, ferroptosis, senescence, and nuclear receptors. Clinical interventions targeting these pathways have shown promising results in addressing liver inflammation and fibrosis, as well as in modulating glucose and lipid metabolism and metabolic stress responses. Therefore, a comprehensive understanding of HSC phenotypes and metabolic mechanisms presents opportunities for novel therapeutic approaches aimed at halting or even reversing chronic liver diseases.

## Introduction

Hepatic stellate cells (HSCs) constitute approximately 15% of the total liver intrinsic cell population and about 30% of non-parenchymal cells. HSCs are not only the primary cell type involved in fibrosis during liver injury but also play a crucial role in liver regeneration and cancer progression 1. Under normal conditions, HSCs exist in a quiescent state, do not express α-smooth muscle actin (α-SMA), and exhibit low levels of proliferation and collagen synthesis 2. However, when the liver is subjected to inflammation or mechanical injury, HSCs become activated, and their phenotype transitions from quiescent to activated. Currently, HSCs are recognized for their high plasticity, which allows them to regulate energy and nutrient balance, inflammatory responses, immune functions, and liver growth in their activated state 3. These processes rely on precise regulation of energy consumption and metabolic adaptability 4. Recent studies have provided insights into the mechanisms governing HSC metabolism and their roles in liver homeostasis and the response to damage 5. Given that the liver plays a pivotal role in regulating its metabolic processes, HSCs are also susceptible to disruptions in systemic metabolic regulation. Furthermore, HSCs serve as a model for metabolic homeostasis beyond the liver, primarily because they are liver-specific pericytes, akin to cells with fibrotic potential in other organs. Importantly, HSCs are an often-overlooked determinant of immune metabolism that supports liver function and inflammatory responses 6.

Chronic liver injury, primarily due to metabolic dysfunction-associated steatotic liver disease (MASLD), metabolic dysfunction-associated alcoholic liver disease (MetALD), or viral hepatitis, can lead to liver fibrosis, cirrhosis, and hepatocellular carcinoma 7. Chronic liver damage promotes HSC activation, resulting in the accumulation of extracellular matrix proteins that disrupt the liver's architecture and impair its functionality 8. Various signals that indicate cellular damage trigger HSC activation. These signals include pro-inflammatory cytokines produced by infiltrating immune cells, apoptotic bodies from hepatocytes, growth factor activation mediated by endothelial cells, and an increased burden of reactive oxygen species (ROS) 9. Numerous paracrine and autocrine signaling loops, including fibrogenic signals such as transforming growth factor beta 1 (TGF-β1) and connective tissue growth factor (CTGF), amplify the response of HSCs to liver damage 10. Besides, recent research has found that targeting Wnt/β-catenin signaling and lactate dehydrogenase A and targeting YAP has therapeutic promise for liver fibrosis through mediated HSC glycolysis, death susceptibility and senescence 11. Single-cell RNA sequencing analyses reveals a high heterogeneity characterization and tightly interrelated network of HSCs and macrophage subpopulations in liver fibrosis 12.

This review has revealed that multiple cells are involved in the activation of HSC in chronic liver injury, mainly including hepatocytes, liver sinusoidal endothelial cells (LSECs) 13, platelets 14, and some immune cells such as macrophages, neutrophils, and various innate lymphocytes and unconventional T cell (UTC) populations 15. This situation influences immunological processes by releasing chemokines and cytokines, or transdifferentiate into myofibroblasts that produce matrix 16, 17. Changes in HSC phenotypes and functions include considerable modifications in transcriptional and protein synthesis, requiring metabolic adjustments in the metabolism of cellular substrates, such as glucose and lipid metabolism, and bearing similarities to the alterations linked to the Warburg effect in cancer cells 18, 19. The activation of HSCs and their alterations in metabolism are also regulated by nuclear receptor signaling and metabolic stress responses. These changes in metabolism are essential for the inflammatory and fibrogenic activation of HSCs 20. Therefore, altering these pathways may present chances for cutting-edge treatment strategies to stop or even reverse the advancement of chronic liver injury.

## The function of HSCs in chronic livers diseases

Under healthy condition, HSC is located within the Disse space, closely adhering to the LSECs and hepatocytes. They constitute approximately 10% of all resident liver cells 21. Under chronic liver diseases, activated HSCs express emerging molecular markers and release cellular signals that influence the critical cellular response to chronic liver damage^22^. The terms of 'initiation' and provide a useful framework for understanding the progression of HSC activation in chronic liver diseases. 'Initiation' means to early events that prompt cells to respond to a wide array of extracellular signals. The hallmarks of initiation include the rapid activation of growth factor receptors, the development of a contractile and fibrogenic phenotype, and the modulation of growth factor signaling. Events that further enhance the active phenotype are indicative of 'perpetuation' in chronic liver diseases, especially MASLD and MetALD 23, 24.

**Figure 1:**
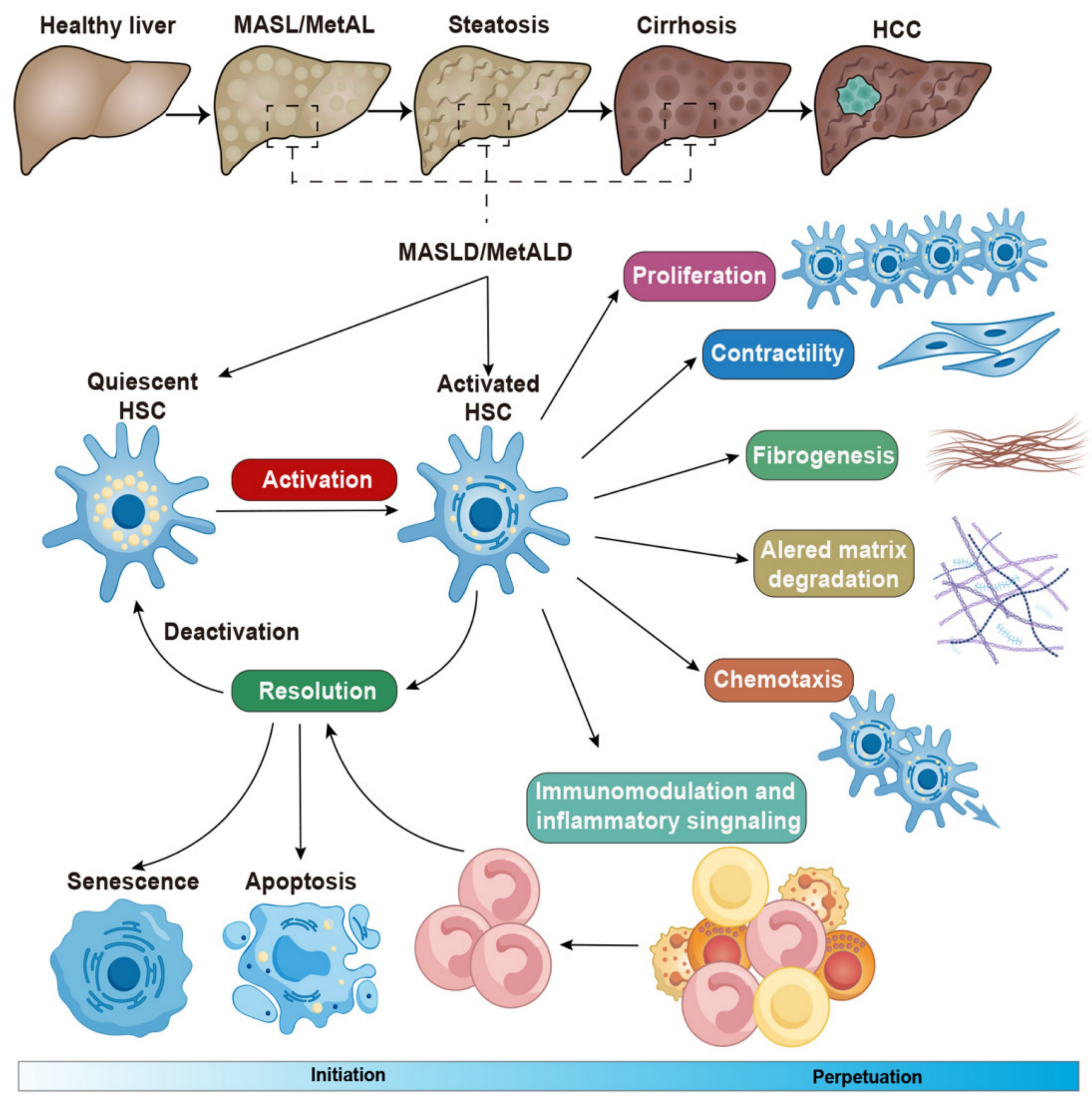
The initiation and perpetuation of HSCs in chronic liver diseases. Conversion of quiescent HSCs to their activated state is triggered by in chronic liver diseases. This activation is characterized by distinct phenotypic changes, including fibrogenesis, increased contractility, proliferation, altered matrix degradation, chemotaxis, and enhanced immunological and inflammatory signaling. During the resolution of hepatic fibrosis, activated HSCs can be eliminated through three mechanisms: apoptosis, senescence, or reversion to an inactivated state. Adapted with permission from 35, Copyright 2021 Cell Press.

HSCs can be activated by initial stimuli during chronic liver diseases, such as a high-fat diet and excessive alcohol consumption, which may trigger to multiple receptors and factors. For instance, lymphocytes, monocytes, Kupffer cells, and damaged hepatocytes release TGF-β during the development of MASLD and MetALD 25, 26. Under these conditions, neutrophils and T helper (T_H_)17 cells produce interleukin (IL)-17, which activates the TGF-β receptor II (TGF-βRII) and increases HSC susceptibility to TGF-β signaling 27, 28. The αV subunits of integrins activate TGF-β in the extracellular matrix, leading to the contraction of activated HSCs and contributing to the development of liver fibrosis 29, 30. Both endothelial cells and macrophages can release platelet-derived growth factor (PDGF), which binds to the platelet-derived growth factor receptor β (PDGFRβ) and promotes the activation of HSCs in in hepatic steatosis and fibrosis 31. In the development of MASLD, steatotic hepatocytes produce lipid mediators, P2Y_14_ ligands, and extracellular vesicles, all of which contribute to HSC activation 32-34. Collectively, these factors lead to increased proliferation, contractility, fibrogenesis, matrix degradation, and immune and inflammatory signaling, ultimately contributing to the formation of scars in chronic liver diseases (**Figure 1**).

The above activation of quiescent HSCs contributes to fibrosis formation in chronic liver diseases, through their transdifferentiation into highly proliferative, extracellular matrix-producing activated hepatic stellate cells or myofibroblasts 35. Once activated, HSCs lose intracellular fatty acids but the role of fatty acid oxidation, and result in steatosis in liver diseases 36. Besides, these cells regulate hepatic immune homeostasis and inflammation, thereby contributing to worsening liver injury, mainly through the mechanisms of crucial signaling pathways, such as toll-like receptor (TLR) receptor, TGF-β and hedgehog mediated hepatic injury 16. The activation of HSCs also affects oxidative stress in liver damage, result in tissue injury and trigger endoplasmic reticulum stress by the overproduction of proteins 37.

**Figure 2:**
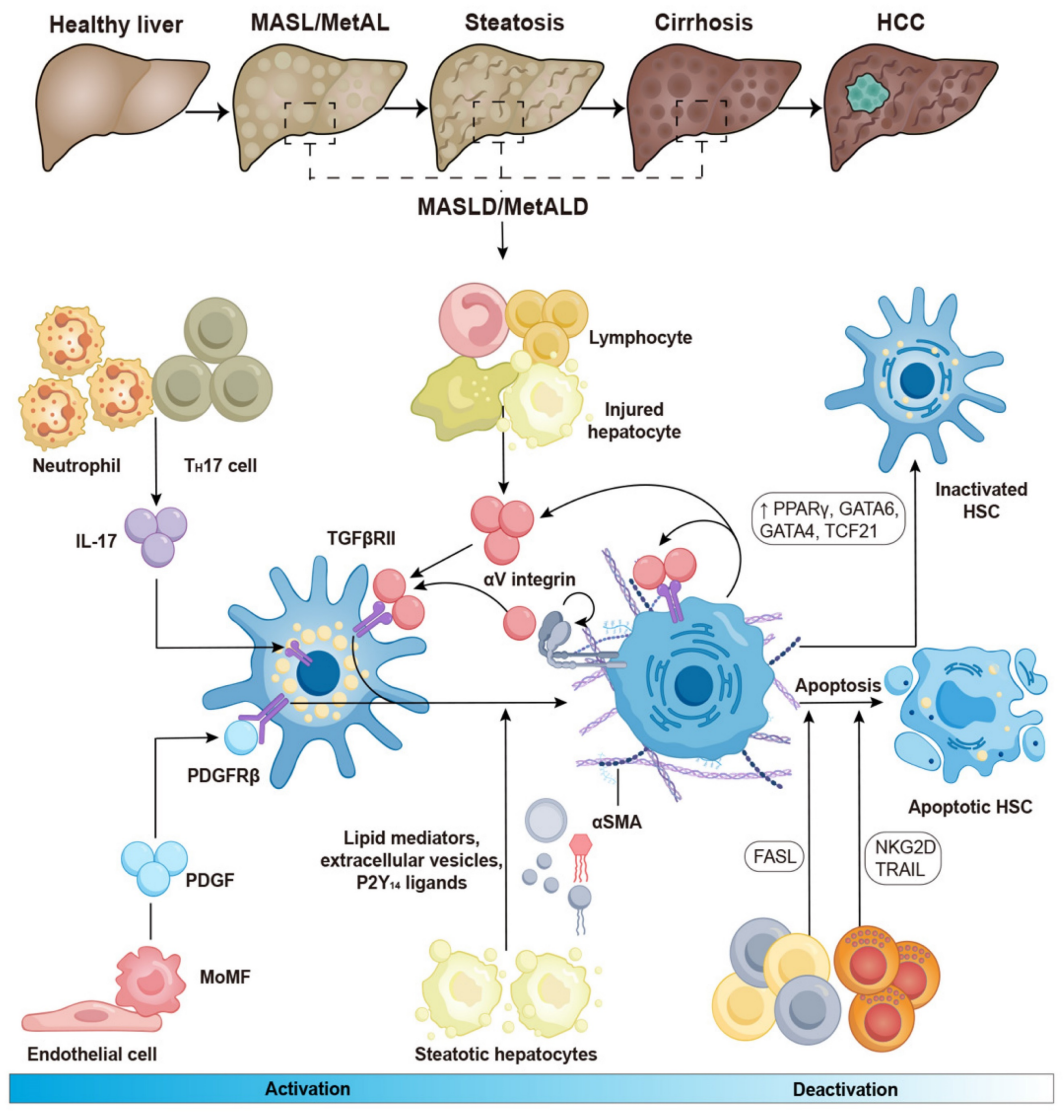
The activation and deactivation of HSCs in chronic liver diseases. When profibrotic stimulation in chronic liver diseases activates HSCs, quiescent HSCs convert into myofibroblasts. This transformation results in a decrease in their vitamin A content, stimulation of α-SMA, and synthesis of collagen type I. The conversion of HSCs is induced by TGF-β, which is produced by infiltrating lymphocytes, monocytes/macrophages, and damaged hepatocytes. TGF-βRII is upregulated by IL-17, which is generated by T_H_17 cells and neutrophils, thereby increasing HSC sensitivity to TGF-β stimulation. When latent TGF-β binds to ECM proteins, it becomes inactive but can be released when activated HSCs contract through the action of αV integrin. In a feed-forward cycle, activated HSCs perpetuate their activation by producing TGF-β. HSC activation is also stimulated by PDGF, which is secreted by macrophages and endothelial cells. Additionally, HSC activation is sustained by lipid mediators, P2Y_14_ signaling, and extracellular vesicles released by injured hepatocytes. Following the resolution of fibrosis, HSCs undergo either apoptosis or revert to an inactive state, a process mediated by the overexpression of transcription factors such as TCF21, GATA4, GATA6, and PPARγ. Lymphocytes, including NK cells, γδ T cells, and CD8^+^ T cells can effectively eliminate activated HSCs and myofibroblasts by inducing apoptosis. FASL, Fas ligand; GATA 4/6, GATA-binding factor 4/6; IL-17, interleukin-17; MoMFs, monocyte-derived macrophages; NKG2D, NK receptor group 2 member D; PDGF, platelet-derived growth factor; PDGFRβ, platelet-derived growth factor receptor β; PPAR, peroxisome proliferator-activated receptor; α-SMA, α-smooth muscle actin; TCF21, transcription factor 21; TGF-βRII, TGF-β receptor II; T_H_ 17, T helper 17; TRAIL, tumour necrosis factor-related apoptosis-inducing ligand. Adapted with permission from 8, Copyright 2023 Springer Nature.

Different states of HSC perpetuation that distinguish between an early fibrogenic phenotype and a later inflammatory senescent phenotype are now integral to a more comprehensive understanding of the phase involved in the development of liver diseases 38. Several molecules and signals can influence the progression of liver disease through HSC apoptosis, senescence, and reversion to a more quiescent, inactive state—three primary processes that contribute to HSC elimination 39, 40. For example, liver γδ T cells and CD8^+^ T cells utilize the FAS-FAS ligand pathway to induce HSC apoptosis, while natural killer (NK) cells depend on NKG2D and tumor necrosis factor (TNF)-related apoptosis-inducing ligands to eliminate HSCs in chronic liver diseases 41. Furthermore, the expression of peroxisome proliferator-activated receptor (PPAR)γ, GATA-binding factor 6 (GATA6), GATA4, and transcription factor 21 (TCF21) appears to play a role in regulating HSC reversion to an inactive state in liver fibrosis (Figure 2) 42-44.

## The mechanism of HSC activation in chronic liver diseases

Under chronic liver diseases, multiple cell injury occurs, including hepatocyte cell death, LESCs injury and stimulation of immune cells. These cells secrete pro-inflammatory cytokines and chemokines, which ultimately activate HSCs. Then, the activation of quiescent HSCs leads to their transdifferentiation into highly proliferative, extracellular matrix-producing activated HSCs or myofibroblasts, which are key factors in progression of chronic liver diseases.

### Multiple cells types influence HSC activation

**Figure 3:**
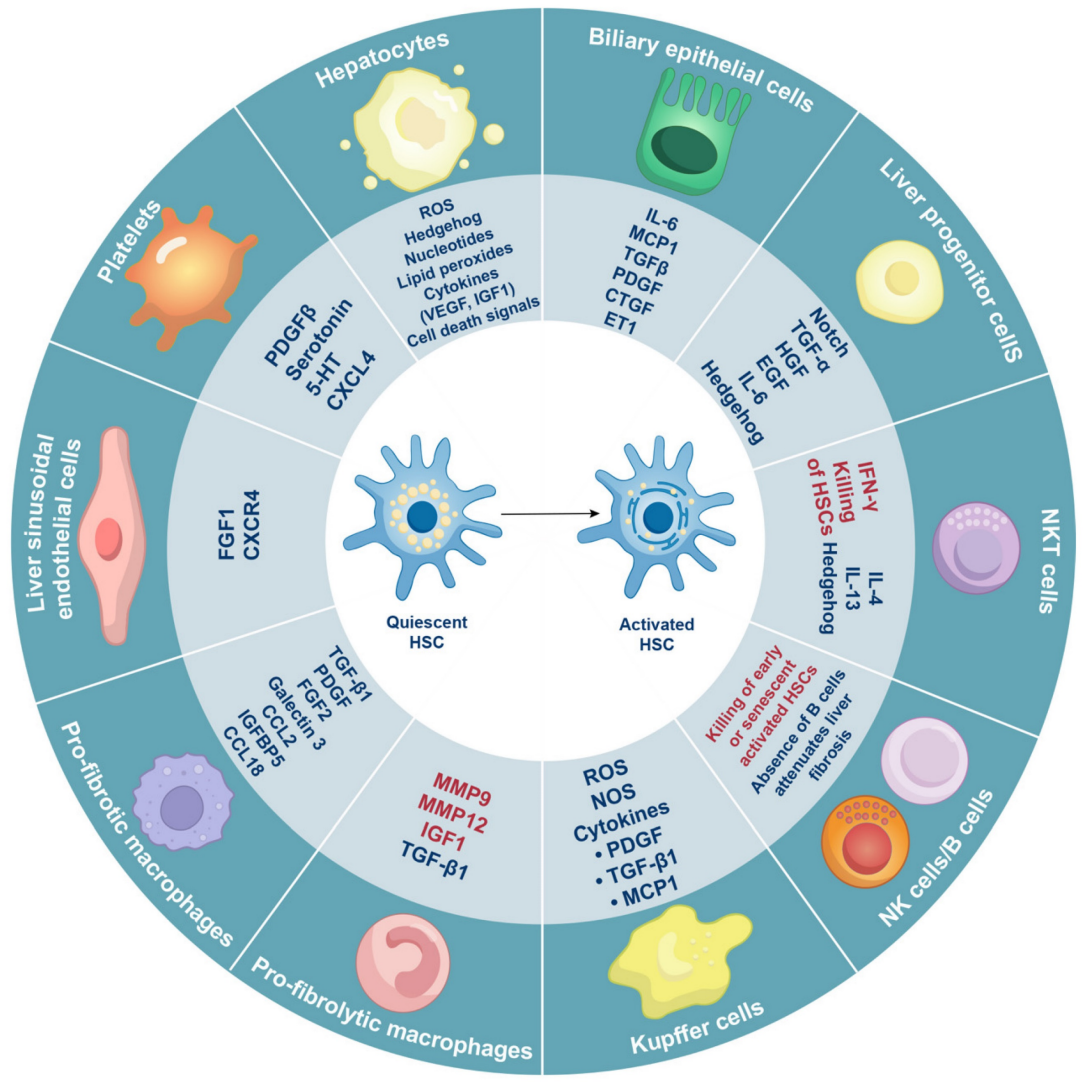
Multiple cell types influence the activation of HSCs in chronic liver diseases. Hepatocytes, macrophages, biliary epithelial cells, liver progenitor cells, LESCs, NK cells, NKT cells, platelets, and B cells can either stimulate (indicated in blue font) or inhibit (indicated in red font) HSC activation by releasing various hormones, cytokines, and other signaling molecules. CCL2/18, C-C motif ligand 2/18; CTGF, connective tissue growth factor; CXCL4, C-X-C motif ligand 4; CXCR4, C-X-C chemokine receptor 4; EGF, epidermal growth factor; ET1, endothelin-1; FGF1/2, fibroblast growth factor 1/2; HGF, hepatocyte growth factor; 5-HT, 5-hydroxytryptamine; IFN-γ, interferon-γ; IL-6/4/13, interleukin-6/4/13; IGF1, insulin-like growth factor 1; IGFBP5, insulin-like growth factor-binding protein-5; MCP1, monocyte chemoattractant protein 1; MMP9/12, Matrix metalloproteinase-9/12; PDGF, platelet-derived growth factor; ROS, reactive oxygen species; TGF-α/β, transforming growth factor-α/β; VEGF, vascular endothelial growth factor.

Even during homeostasis, liver tissue comprises a diverse array of cell types that are distributed throughout the parenchyma, with a higher concentration in the periportal regions. These cell types include hepatocytes, macrophages, biliary epithelial cells, liver progenitor cells, LESCs, NK cells, natural killer T (NKT) cells, platelets, B cells, mucosal-associated invariant T (MAIT) cells, γδ T cells, neutrophils, mast cells, and innate lymphoid cells. These various cell types converge on HSCs to promote HSC activation (**Figure 3**).

#### Hepatocytes affecting HSC activation

The destruction of hepatocytes occurs under certain conditions such as metabolic dysfunction-associated steatohepatitis (MASH) and viral infections, both of which are linked to parenchymal liver injury 45. This process is facilitated by an expanding array of mediators, including nucleotides, hedgehog ligands, and reactive oxygen species (ROS) 46. Damage-associated molecular patterns (DAMPs) secreted by dying hepatocytes can promote the activation of HSCs either directly or indirectly 47. NACHT, LRR and PYD domains-containing protein 3 (NLRP3) is a crucial component of the inflammasome and acts as a downstream regulator of DAMPs 48. Hepatocyte pyroptosis leads to the release of complex inflammatory particles, including the NLRP3 inflammasome, from within hepatocytes into the extracellular space. Mice with a constitutively active mutant NLRP3 exhibit severe liver inflammation, pyroptotic hepatocyte death, and HSC activation, which is accompanied by fibrosis 49. In response to liver damage, hepatocytes produce IL33, which activates and expands liver-resident innate lymphoid cells (ILCs) 50. The three known subsets of ILCs are ILC1, ILC2, and ILC3, with ILC2 being responsible for mediating liver damage that leads to HSC activation. Furthermore, the engulfment of apoptotic hepatocyte bodies by HSCs promotes their activation as the phagocytosis of these apoptotic bodies can enhance HSC activation 51.

#### Monocytes and macrophages affecting HSC activation

**Figure 4:**
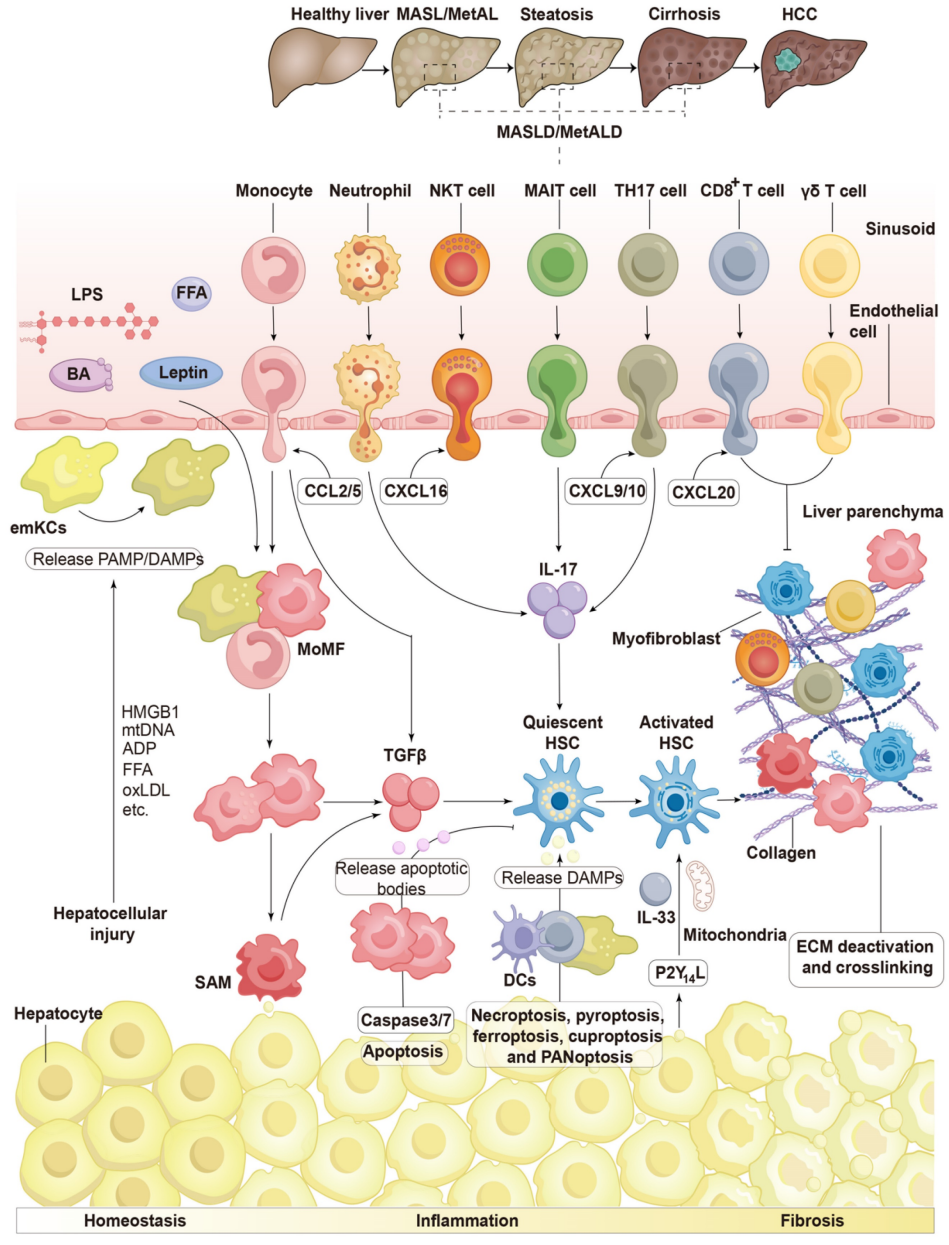
HSC activation is influenced by immune cells in chronic liver diseases. Chronic liver inflammation is initiated by hepatocellular damage, which releases DAMP and PAMP signals, including HMGB1, mtDNA, ADP, FFA, oxLDL, and others. These signaling molecules, along with cytokines and chemokines produced by activated emKCs, attract immune cells from the bloodstream, particularly resident macrophages to the liver, leading to significant phenotypic alterations. These immune cells respond promptly to disruptions in extrahepatic signals, primarily BAs and bacterial products such as LPS from the intestine, as well as lipid mediators like FFAs and leptin from adipose tissue and damaged hepatocytes, resulting in a pro-inflammatory response. Monocytes, driven by CCL2, are the first immune cells to arrive in the liver following injury. They release profibrogenic molecules, including TGF-β, and differentiate into MoMFs, which subsequently prolong the inflammatory response. MoMFs may further differentiate into SAM, which produce TGF-β and activate HSCs. Neutrophils may arrive early during the inflammatory response and promote fibrogenesis by producing IL-17, which stimulates HSCs. Auto-aggressive CD8^+^ T lymphocytes accelerate hepatocyte destruction. Injured hepatocytes emit danger signals, such as P2Y_14_L and alarmins, which include IL-33 and mitochondrial metabolites, potentially activating HSCs. Lymphocytes are attracted by various chemokines, including CXCL16 released by NKT cells, CXCL9 and CXCL10 released by conventional T cells, and CCL20 released by γδ T cells. TH17 cells and MAIT cells produce IL-17, which promotes fibrogenesis. However, certain CD8^+^ T cells, γδ T cells, and NK cells inhibit fibrosis formation by inducing apoptosis in myofibroblasts. The activation of caspase-3/7 in apoptotic hepatocytes leads to the production of apoptotic bodies, which can activate HSCs directly or indirectly through macrophage activation. DAMPs, particularly HMGB1, can be released through necroptosis, pyroptosis, ferroptosis, cuproptosis, and PANoptosis, causing macrophages, monocytes, and DCs to aggregate, secrete inflammatory factors, and further amplify the inflammatory response. ADP, adenosine diphosphate; BA, bile acid; CCL2/5, C-C motif ligand 2/5; CXCL, C-X-C motif ligand16; DCs, dendritic cells; DAMPs, damage-associated molecular patterns; ECM, extracellular matrix; emKCs, embryonic KCs; FFA, free fatty acids; HMGB1, high-mobility group box 1; HSCs, hepatic stellate cells; IL-33, interleukin-33; LPS, lipopolysaccharide; MAIT, Mucosal-associated invariant T; MoMF, monocyte-derived macrophage; NKT, natural killer T; oxLDL, oxidized low-density lipoprotein; PAMP, pathogen-associated molecular pattern; SAM, scar-associated macrophage; TGF, transforming growth factor; T_H_17, T helper 17. Adapted with permission from 8, Copyright 2023 Springer Nature.

Hepatic monocytes can be broadly categorized into macrophages, which primarily include tissue-resident Kupffer cells and monocyte-derived macrophages (MoMFs) (**Figure 4**) 52. Selective depletion of macrophages in mice, results in CD11b-diphtheria toxin transgenic mice, leads to reduced activation of HSC and fibrogenesis following liver injury induced by CCl_4_. This suggests that the removal of macrophages during sustained injury leads to a decrease in liver damage 53. The recruited monocytes consist of two subsets: CD11c ^(-)^/Ly6C ^(+)^ cells and CD11c ^(+)^/Ly6C^ (-)^ cells. Ly6C ^(+)^ cells are inflammatory and recruited macrophages that contribute to advanced liver injury, while Ly6C^ (-)^ cells are known as 'alternative macrophages' that inhibit inflammatory responses and promote HSC activation. Ly6C^ (+)^ monocytes exhibit significantly higher levels of CC chemokine receptor (CCR)1 and CCR2, whereas Ly6C ^(-)^ cells show elevated expression of CCR5 and CX3C chemokine receptor 1 (CX3CR1). CCR1 primarily stimulates HSC activation by bone marrow-derived macrophages 54. In models of bile duct ligation and CCl_4_ therapy, mice lacking CCR1 demonstrate decreased macrophage infiltration. Intrahepatic Ly6C^ (+)^ monocyte-derived macrophages are recruited in a CCR2-dependent manner during fibrogenesis triggered by CCl_4_ injection, indicating that CCR2 plays a crucial role in the accumulation of these macrophages. In CCR2-deficient mice, the recruitment of damaged Ly6C^ (+)^ monocytes is diminished, leading to decreased activation of HSC 55, 56. Furthermore, in CCR2-deficient mice, a choline-deficient, L-amino acid-defined diet results in reduced infiltration of Ly6C^ (+)^ macrophages, consequently leading to less liver injury 57.

Kupffer cells represent the largest resident macrophage population in the body and play a critical role in activating HSCs 58. Bone marrow-derived monocytes (BMDMs) migrate to the damaged liver, where they promptly respond to inflammatory signals and differentiate into macrophages 59. In the context of inflammation, a significant influx of BMDMs is responsible for producing MoMFs, which, in turn, expand the pool of liver macrophages. Although MoMFs and Kupffer cells differ in terms of phenotype and function, mouse models demonstrate that the two lineages exhibit remarkable plasticity 60. Extensive research utilizing single-cell RNA sequencing has provided unprecedented insights into the heterogeneity of hepatic myeloid cells. One important finding is that MoMFs can replace Kupffer cells and adopt the phenotype of lipid-associated macrophages (LAMs) or scar-associated macrophages (SAMs), characterized by the expression of triggering receptor expressed on myeloid cells 2 (TREM2), CD9, and osteopontin, which may stimulate HSC activation 61, 62. Spatial proteogenomics analyses have confirmed the persistence of these macrophage subtypes across various species, and single-cell RNA sequencing analyses conducted on human liver tissues have identified SAMs as a distinct group located in the fibrotic niche of cirrhotic livers, although many findings are still primarily based on mouse models. Notably, a hallmark of severe fibrosis in human liver biopsy samples across different etiologies is the accumulation of inflammatory MoMFs in portal regions, particularly surrounding ductular responses 63.

#### LSECs affecting HSC activation

In a healthy liver, the differentiation of LSECs inhibit HSC activation and promotes the transition of active HSCs to a quiescent state through the generation of nitric oxide (NO) derived from vascular endothelial growth factor (VEGF) 64. Capillarization diminishes the ability of LSECs to inhibit HSC activation and is characterized by a lack of LSEC differentiation following liver injury 65. In rats with thioacetamide-induced cirrhosis, the restoration of capillarization through the injection of BAY 60-2770, a soluble guanylate cyclase stimulator, leads to HSC quiescence. Endothelial nitric oxide synthase acts as the catalyst for the production of NO, which is the mechanism by which VEGF functions to activate soluble guanylate cyclase 66.

Given the circumstances surrounding liver damage, liver regeneration and fibrosis may be influenced by LSECs 67. The differential expression of stromal cell-derived factor 1 receptors, C-X-C chemokine receptor (CXCR) 7, and CXCR4 following chronic liver damage indicates that angiocrine signals from LSECs promote HSC activation and regeneration immediately after injury. CXCR7 regulates regenerative pathways by inducing the expression of ID1, a known inhibitor of DNA-binding protein 1 68. Additionally, fibroblast growth factor receptor 1 (FGFR1) and CXCR4, which provide paracrine stimulation to HSCs, are key drivers of HSC activation and liver injury processes 69. Regenerative pathways are restored in mice with LSEC-specific depletion of FGFR1 or CXCR4. The depletion of either FGFR1 or CXCR4 in LSECs uniquely facilitates the recovery of liver regenerative pathways in these mice 70.

#### Platelets affecting HSC activation

Platelets are a crucial cellular source of PDGFβ and TGF-β, which activate HSCs and influence various chronic liver diseases, including MASH, MetALD and hepatitis B and C 71, 72. Platelets produce PDGFβ to activate HSCs and facilitate liver injury in multidrug resistance protein 2 (MDR2)/Abcb4-null mice, a model that promotes advanced biliary fibrosis. Depleting platelets or selectively inhibiting PDGFβ may reduce biliary fibrosis in patients with liver diseases 73. Silencing Nogo-B suppresses the proliferation, fibrosis, and autophagy of HSCs while inducing cell cycle arrest and senescence of HSCs through the PDGFβ 74. Gomisin D can inhibit HSC proliferation and activation, promote HSC apoptosis, and alleviate CCl_4_-induced liver injury by targeting PDGFRβ and regulating PDGFRβ signaling pathway 75.

#### Dendritic cells affecting HSC activation

DCs are a specialized type of antigen-presenting cell that primarily including plasmacytoid dendritic cells (pDCs) and conventional (classical) dendritic cells (cDC1s and cDC2s) 76. Although mature DCs are enlarged in damaged liver tissue, the precise mechanisms by which they contribute to the activation of HSCs remain unclear. Several studies in murine models have demonstrated that inhibiting or depleting DC maturation and function reduces inflammation and, consequently, inhibits HSC activation and liver injury 77, 78. However, other research has indicated that a reduction in DCs has no effect on liver damage 79. Furthermore, in murine models of liver fibrosis, DCs have been shown to restrict inflammation and fibrogenesis. Specifically, cDC1s, particularly those expressing the CD103 ^(+)^ subtype, have been identified as a protective subset in mice with metabolic diseases 80. More recent studies, however, suggest that cDC1s may also play a detrimental role in liver diseases 81. These cells are widely distributed in humans with metabolic dysfunction-associated fatty liver disease (MAFLD) and in mice with steatohepatitis, and their presence correlates with fibrosis scores and disease severity 82. In murine models, X-C motif chemokine receptor 1-expressing cDC1s exacerbate liver disease by inducing inflammatory reprogramming of CD8^+^ T cells 83.

#### T and B lymphocytes affecting HSC activation

In a healthy liver, T and B cells predominantly reside along the portal tracts. However, they often infiltrate the parenchyma during inflammatory responses 84. In liver disorders triggered by antigens, such as autoimmune hepatitis and chronic viral hepatitis, adaptive immune cells play a crucial role 85.

For example, in chronic hepatitis B virus infection, T cell dysfunction is often attributed to insufficient priming of CD8^+^ T cells by hepatocytes. This process can be reversed through Kupffer cell-mediated cross-presentation in mice. While these inflammatory events contribute to the formation of fibrosis through their cytotoxic effects on hepatocytes, adaptive immunity may also influence hepatic fibrosis independently of specific antigens. This non-specific activation is particularly relevant to MAFLD, as it is driven by mediators released during metabolic processes linked with liver injury. These mediators primarily include acetate and extracellular adenosine 5' triphosphate (ATP), which may activate liver-resident CD8^+^ T cells characterized by high expression of CXCR6, PD-1, and perforin 86. Consequently, these T cells perpetuate auto-aggression against hepatocytes in an MHC class I-independent manner, thereby accelerating the progression of steatohepatitis 87.

There is conflicting data regarding the role of CD8^+^ cytotoxic T cells in liver damage. This discrepancy may be attributed to a combination of auto-aggressive and protective T cell immunity 88. The absence of CD8^+^ T cells has no effect on fibrosis formation in a mouse model of toxic liver fibrosis 89. However, the activation of splenic CD8^+^ T cells in the same context exacerbates disease progression 90. Conversely, in a mouse model of MASH induced by obesity, a reduction in CD8^+^ T cells leads to decreased activation of HSC and reduced liver inflammation. In this scenario, HSC may be directly activated by CD8^+^ T cells 91. Furthermore, studies have shown that tissue-resident CD8^+^ T lymphocytes with a memory phenotype facilitate the resolution of hepatic fibrosis by inducing apoptosis in activated HSC. The adoptive transfer of CD8^+^ T lymphocytes inhibits the progression of fibrosis, while their absence hinders the resolution of fibrosis 92. These contradictory findings suggest that the development and resolution of hepatic fibrosis may involve distinct populations of CD8^+^ T cells.

T_H_ cells, including T_H_2 and T_H_17 cells, are closely associated with liver injury, particularly in the development of fibrosis 88, 93. Numerous profibrogenic cytokines, such as IL-4 and IL-13, are released by T_H_2 cells, which activate fibroblasts and promote extracellular matrix remodeling 94. In contrast, the cytokines IFN-γ and IL-12 produced by T_H_1 cells exhibit a somewhat antifibrotic effect 88. T_H_17 cells contribute to liver fibrosis by producing IL-17 and IL-22, which stimulate the production of TGF-β in the liver, enhance TGF-β signaling in HSCs, and promote collagen and pro-inflammatory cytokine production by HSCs and Kupffer cells 95. These processes ultimately lead to the progression of liver fibrosis. Furthermore, fibrosis formation is inhibited in mice lacking the IL-17 or IL-22 receptor, and the advancement of fibrosis can be halted *in vivo* by depleting IL-17 96. Notably, IL-22 may have protective effects against liver damage. Through the induction of HSC senescence, IL-22 agonism reduces liver fibrosis in mice treated with CCl_4_, protects hepatocytes from damage during acute hepatitis, and alleviates hepatic inflammation in mice with acute-on-chronic liver failure 97. In a phase II clinical study, the IL-22 agonist F-652 has been shown to enhance hepatic regeneration and reduce hepatic inflammation in 18 individuals with severe alcoholic hepatitis 98.

Regulatory T (T_reg_) cells may prevent liver injury by releasing IL-10. As an anti-inflammatory cytokine, IL-10 reduces collagen I production, inhibits the activation of Kupffer cells, and decreases IL-17 production by T_H_17 cells, ultimately leading to reduced activation of HSCs 99. Mice deficient in IL-10 are consistently more susceptible to liver injury 100. Conversely, T_reg_ cells may hinder the resolution of fibrosis in mice by suppressing the synthesis of matrix metalloproteinases by Kupffer cells, which contributes to the persistence of fibrosis 101.

B cells constitute only 5% of intrahepatic lymphocytes in a healthy human liver, representing a modest fraction of liver immune cells 102. For example, mice lacking B cells do not develop fibrosis in either toxic or diet-induced models, but B cells that accumulate in the damaged liver exhibit an activated state and release pro-inflammatory cytokines, which promotes HSC activation. Data from individuals with MAFLD and mouse models indicate that gastrointestinal B cells may contribute to liver injury by secreting immunoglobulin A and activating Fcγ receptor signaling on myeloid cells 103. Interestingly, certain types of B cells, such as regulatory B cells that produce IL-10, may also provide protection against HSC activation and inflammation associated with MAFLD 104.

#### Unconventional T cells affecting HSC activation

UTCs are a diverse collection of lymphocytes that include NKT cells, MAIT cells, and γδ T cells 105-108. NKT cells appear to play dual roles in chronic liver damage. They can exacerbate liver injury through the production of profibrotic cytokines, such as IL-4 and IL-13, which may activate HSCs 109. Under another conditions, NK cells may eliminate HSCs by expressing death receptor ligands, including Tumor Necrosis Factor-Related Apoptosis-Inducing Ligand and Fas Ligand (also known as TNF ligand superfamily member 6), while simultaneously mitigating liver injury by producing IFN-γ. In addition to their direct influence on liver injury, NKT cells can polarize into profibrotic T_H_2 and T_H_1 cell responses by secreting cytokines like IL-4 and IFN-γ, which inhibit the activation of HSCs 110. Furthermore, NK cells assist in the clearance of senescent activated HSCs, facilitating the resolution of fibrosis 111. NKT cells exhibit a range of complex, context-dependent roles in liver fibrosis and HSC activation. Similar to NK cells, NKT cells produce IFN-γ and promptly eliminate activated HSCs, alleviating liver damage 112. However, NKT cells also display profibrogenic characteristics. The migration of NKT cells is regulated by CXCR6, which is expressed by NKT cells, and its ligand, C-X-C motif ligand (CXCL) 16, produced by endothelial cells and macrophages. Mice deficient in CXCR6 demonstrate protection against liver injury, but the progression of liver injury resumes following the adoptive transfer of NKT cells into CXCR6-deficient mice 113.

The role of MAIT cells in the activation of HSCs remains incompletely understood. Recent research suggests that MAIT cells exhibit profibrogenic activities in chronically damaged livers. They can stimulate HSC activation through IL-17 or promote the pro-inflammatory polarization of macrophages. Studies have shown that the absence of MAIT cells inhibits the activation of HSCs and fibrosis formation in mice 114. Functionally, MAIT cells have been proposed as the human equivalent of NKT cells 115.

γδ T cells have been shown to proliferate in these conditions. However, there is limited information regarding their specific role in the activation of HSCs and the development of chronic liver diseases 88. γδ T cells exert profibrogenic effects by increasing the production of hepatic IL-17 in mice with liver fibrosis, which promotes the activation of HSCs. Additionally, γδ T cells induce apoptosis in activated HSCs and enhance NK cell-mediated cytotoxicity against these activated HSCs, thereby alleviating liver damage 116.

#### Neutrophils and mast cells affecting HSC activation

Neutrophils are present in various forms of chronic liver disease. By producing inflammatory cytokines, activating Kupffer cells, and attracting other immune cells, neutrophils can exacerbate hepatic inflammation. However, they do not appear to have a direct impact on the activation of HSCs 117. Research conducted in mice suggests that neutrophils play a crucial role in the activation of HSCs and inflammation by facilitating the functional transition of macrophages from pro-inflammatory to restorative phenotypes. This effect is completely abolished upon neutrophil depletion 118.

Mast cells are innate myeloid cells that play a significant role in allergic reactions and the regulation of host-pathogen interactions in allergies and autoimmune liver diseases 119. In a healthy liver, mast cells are relatively scarce. However, nearly all liver disorders exhibit mast cell infiltration, which appears to primarily contribute to profibrogenic activity 120. The depletion of mast cells has been shown to reduce liver damage, while an increase in mast cell numbers is associated with severe fibrosis and inflammation in mouse models of MAFLD and MASH 121. Similar outcomes have been observed in mouse models of cholestatic liver disease 122. Furthermore, the severity of fibrosis in individuals with viral or alcoholic hepatitis correlates with the number of infiltrating mast cells. One of the key mediators released by activated mast cells is histamine. Given that both histamine inhibition and histamine receptor blockade inhibit the activation of HSCs, reduce liver damage and fibrosis formation in mouse models of cholestatic damage, it appears that histamine signaling plays a crucial role in mast cell-induced inflammation and the development of fibrosis 123.

#### Innate lymphoid cells affecting HSC activation

ILCs comprise several subsets, including lymphoid tissue inducer cells, NK cells, ILC1, ILC2, and ILC3. Hepatic ILCs constitute a significant portion of intrahepatic lymphocytes, primarily consisting of group 1 ILCs, which include NK cells and tissue-resident ILC1s 124. The role of ILCs in the development of chronic liver disease remains an area of ongoing investigation and debate 125. NK cells play a protective role in chronic liver disease in both mice and humans by eliminating activated HSCs, inducing apoptosis, and producing the antifibrotic cytokine IFN-γ. These effects contribute to the resolution of the extracellular matrix and promote a shift towards T_H_1 responses 126. Other studies suggest that the protective effect of NK cells may be highly dependent on the specific NK cell subtype or the stage of disease progression. For instance, NK cells with high expression of CXCR3 appear to be functionally impaired in individuals with chronic hepatitis C virus infection, which subsequently exacerbates chronic liver injury 127. In mice treated with CCl_4_, the antifibrotic effect of NK cells diminishes as the disease advances, potentially due to increased production of TGF-β by HSCs 112, 128.

All of these cell types, including hepatocytes, macrophages, biliary epithelial cells, liver progenitor cells, LESCs, NK cells, NKT cells, platelets, B cells, MAIT cells, γδ T cells, neutrophils, mast cells, and innate lymphoid cells, play a crucial role in chronic liver diseases. These various cell types converge upon HSCs to promote HSC activation, which contribute to release danger signals, extrahepatic signals and chemokines.

### Inflammatory processes influence HSC activation

#### Danger signals released from dying cells

Damaged and dying cells produce soluble mediators that function as alarmins or DAMPs, signaling surrounding cells to respond to chronic liver injury (**Figure 4**) 129. P2Y_14_ ligands have been shown to be released by dying hepatocytes, which interact with the P2Y_14_ receptor on HSCs, leading to their immediate activation in both mice and humans. Consequently, the absence of P2Y_14_ in several preclinical animal models has resulted in reduced liver injury. Injured hepatocytes can release the nuclear protein high-mobility group box 1 (HMGB1), which also promptly stimulates HSCs. Given that hepatocytes contain a high number of mitochondria due to their elevated metabolic activity, mitochondria-derived DAMPs are particularly abundant in the liver. These molecules primarily consist of mitochondrial DNA, which shares similarities with bacterial DNA 130. In preclinical rodent models, mitochondria-derived DAMPs have been shown to stimulate HSC activation and the production of scar tissue. Furthermore, these DAMPs are elevated in patients with MASH and fibrosis 47.

Pattern recognition receptors enable both parenchymal and non-parenchymal cell types in the liver, including HSCs, LESCs, and Kupffer cells, to detect the release of danger signals in chronic liver diseases 131. One significant response to these signals is the production of inflammasomes, which are protein complexes that have been conserved throughout evolution. These complexes are assembled through specific signaling pathways and cellular stress 49. Inflammasomes process IL-1β, release IL-18, and ultimately promote pyroptosis in inflammatory cells, thereby initiating the inflammatory process 132.

IL-33, a cytokine associated with T_H_2 responses, is produced by stressed hepatocytes in both humans and animals suffering from chronic liver diseases 133. The production of IL-33 leads to the recruitment of ILC2s, which subsequently produce IL-13, activating HSCs 134. Furthermore, IL-33 may directly influence HSCs and promote the synthesis of extracellular matrix proteins. In cholestatic liver disorders, bile acids (BAs) accumulate in human and murine tissues, which can trigger inflammation by stimulating the production of pro-inflammatory mediators in hepatocytes and other cell types, including chemokines, cytokines, and adhesion molecules 135. Harmful BAs can be detected by various non-parenchymal liver cells, such as HSCs and macrophages, through the receptor Takeda G protein-coupled receptor 5 (TGR5). This interaction may initiate fibrogenic responses, including cholangiocyte proliferation and ductular reactions 136, 137.

#### Extrahepatic signals

Extrahepatic mediators produced in locations such as the gut and adipose tissue significantly influence liver inflammation. This influence is particularly evident in MAFLD and MASH, where the progression of liver disease is affected by hormones from the gut and adipose tissue, microbiological metabolites, and dietary factors (**Figure 4**) 138, 139. Given the physical and functional interconnection between the intestines and liver, the gut-liver axis can be disrupted in various clinical conditions, serving as an indicator of liver diseases 140. When intestinal homeostasis is compromised, bacterial metabolic products, pathogen-associated molecular patterns (PAMPs), BAs, and nutrients can enter the liver via the portal vein 141. Microbial compounds, such as lipopolysaccharides (LPS), exert a pro-inflammatory effect primarily mediated by liver-resident macrophages and are elevated in numerous liver disorders associated with increased intestinal permeability 142. Additionally, liver inflammation can result from alterations in bacterial metabolites, including ethanol and fatty acid composition 143.

Gut dysbiosis alters the composition of BAs because gut bacteria modify secondary BAs before they are recycled to the liver. Pro-inflammatory microbes outcompete other species in the small intestine when there is a reduction in primary BAs, leading to an increase in toxic BAs, which exacerbates inflammation and damages liver cells 144. Conversely, certain intrinsic or synthetic BAs that activate the farnesoid X receptor (FXR) in hepatocytes suppress the overexpression of inflammation-associated genes and promote cellular longevity 145. Consequently, the effects on hepatic fibrosis and inflammation depend on the specific composition of the BA pool, which is closely linked to metabolic changes 146.

When insulin resistance is present, the flow of free fatty acids from adipose tissue to the liver is accelerated in obese individuals, exacerbating lipotoxicity and subsequent inflammation in chronic liver diseases. This process may be further aggravated by the liver, which can signal adipose tissue to enhance lipolysis 147. Additionally, studies in mice have demonstrated that leptin released from adipose tissue activates liver-resident macrophages and increases their sensitivity to endotoxins, leading to heightened liver inflammation 148. In *in vitro* experiments, leptin-activated Kupffer cells also induce the activation of HSCs, suggesting potential profibrogenic properties 149.

#### Chemokines and chemokine receptors

In chronic liver diseases, chemokines—also known as chemoattractant cytokines—play a crucial role in regulating the recruitment and localization of immune cells. C-C motif ligand (CCL)2, CCL3, CCL5, CXCL9, and CXCL10 are among the first chemokines produced following hepatic injury (**Figure 4**) 150. These chemokines attract monocytes via CCR2 and CCR5, as well as T lymphocytes through CCR5 and CXCR3 151. The CCL2 and CCR2 pathway exerts a profibrogenic effect. CCR2 knockout mice exhibit significantly inactivate HSCs and less fibrosis compared to wild-type mice. Therefore, in animal models of MASH, therapeutic suppression of CCR2 results in a reduction of liver injury and inflammatory responses, while also inhibiting the infiltration of CCR2^+^ monocytes 152. The common profibrogenic roles of CCL3 and CCL5 are supported by observations of decreased immune cell infiltration and alleviation of liver injury in mouse models due to their deficiency or antagonistic interactions 153. In contrast, the fractalkine receptor CX3CR1 limits infiltration and regulates the survival and development of intrahepatic monocytes, thereby mitigating hepatic fibrogenesis *in vivo* 154.

CXCR3 and its ligands, including CXCL9, CXCL10, and CXCL11, play complex roles in the recruitment of various lymphocyte populations 155. In individuals with chronic liver diseases, the levels of CXCL9 and CXCL10, both intrahepatic and peripheral, increase and correlate with the severity of the disease across different stages of fibrosis 156. Preclinical studies have revealed conflicting roles for the CXCR3 axis. Mice lacking CXCR3 are less susceptible to activate HSCs and the induction of steatohepatitis compared to wild-type mice. However, they also exhibit more severe fibrosis following toxic liver injury. The finding that CXCR3 is involved in the recruitment of both pro-inflammatory and anti-inflammatory T cell subsets, such as T_H_1 cells and T_reg_ cells, may help clarify these contradictory effects 157.

CXCR6 and its ligand CXCL16 play a significant role in the recruitment of NKT cells and other lymphocytes during chronic liver disease 158. Mice deficient in CXCR6 demonstrate a reduced accumulation of NKT cells and inactivate HSCs and are protected against the progression of hepatic fibrosis in various experimental models 159. A similar effect on fibrosis can be achieved through the therapeutic suppression of CXCL16, which also decreases intrahepatic levels of pro-inflammatory cytokines and macrophage infiltration 160. However, in animal models, CXCR6^+^ NKT cells exhibit important anticancer properties, highlighting their dual role in chronic liver disorders. Importantly, CXCR6 is not exclusive to NKT cells. It is also present in various lymphocyte subsets, including CD8^+^ T cells that reside in the livers of patients with MASH. These cells contribute to auto-aggression against hepatocytes and display impaired tumor surveillance in MASH-affected mice 161.

CCL20 levels are elevated in mouse models and individuals with chronic liver diseases, correlating with the severity of the condition 162. This chemokine is produced by both parenchymal and non-parenchymal cells and attracts specific γδ T cells via the CCR6 receptor. This interaction prompts activated HSCs to induce apoptosis, thereby limiting fibrosis 116. Additionally, the pathways involving CCR7 and CCL21, as well as CXCR4 and CXCL12, are crucial for the recruitment of immune cells to the damaged liver. Given that these receptors are expressed by various cell types, including lymphocytes, myeloid cells, and stem cells, their precise roles in the disease process are highly context-dependent. However, overexpression of these ligands is frequently observed in numerous hepatic diseases 163.

All these cell types and inflammatory processes converge on HSCs to promote HSC activation, which contributes to the development of chronic liver diseases. After their activation, changes occur in the metabolic mechanisms of the cells themselves.

## Mechanism of metabolic adaptations in activated HSCs

**Figure 5:**
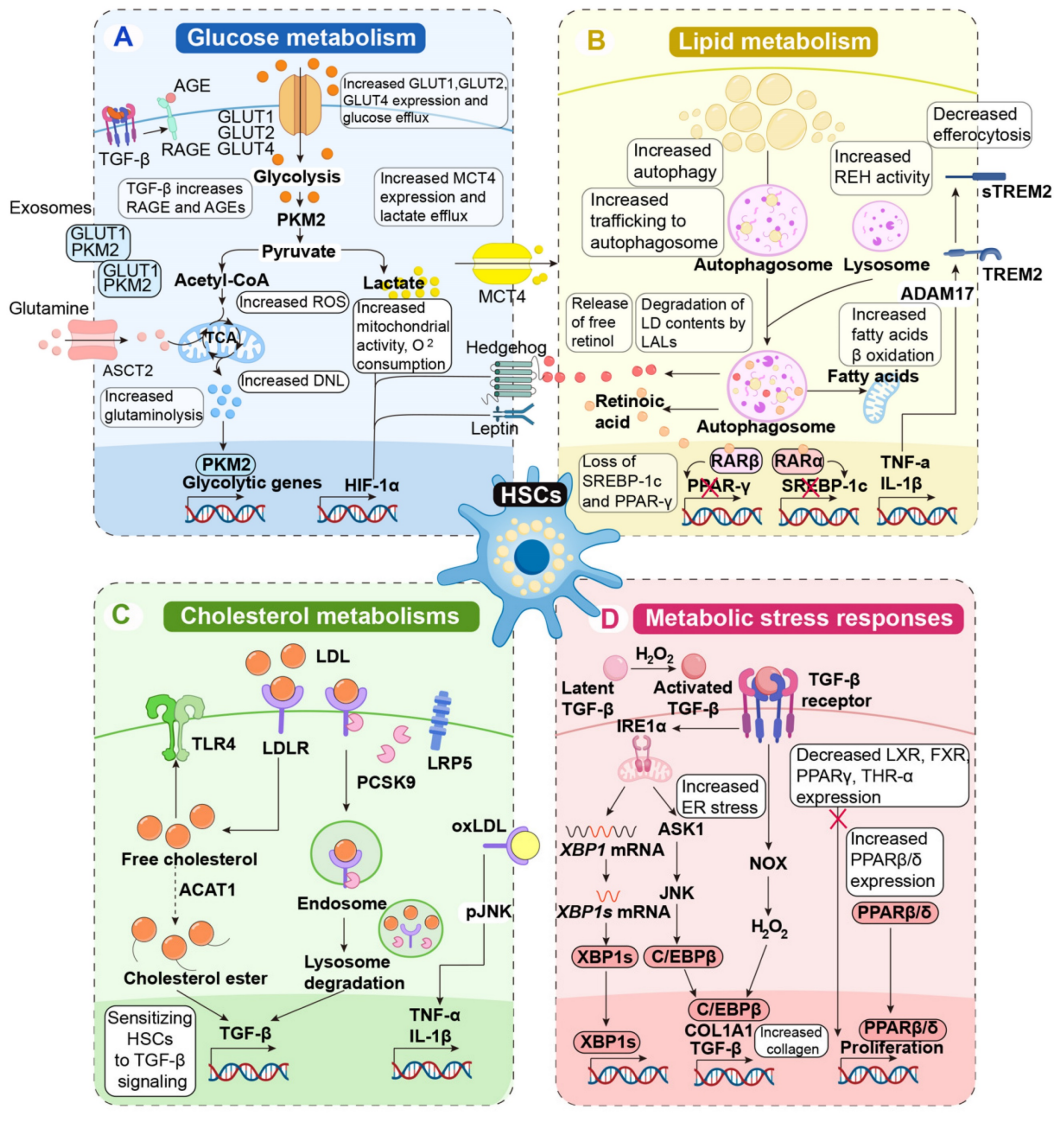
Metabolic reprogramming in fibrogenic HSCs activation in chronic liver diseases. A, HSCs are activated by exosomes containing GLUT1 and PKM2. The overexpression of glucose transporters GLUT1, GLUT2, and GLUT4 facilitates excessive glucose uptake by HSCs, leading to increased glycolysis. Pyruvate, a byproduct of PKM2-catalyzed glycolysis, is largely diverted toward lactate synthesis, resulting in lactate accumulation within HSCs, despite the increased expression of the lactate transporter monocarboxylate transporter 4 (MCT4) and enhanced lactate efflux. In a separate metabolic pathway, pyruvate is converted to acetyl-CoA, which enhances the activity of the TCA cycle and releases lipids that are stored for β-oxidation. PKM2 regulates the expression of metabolic genes, which elevates ROS production and overall mitochondrial activity. Additionally, glutamine enters the cell via the ASCT1 and is metabolized to produce glutamate and α-ketoglutarate, which are subsequently integrated into the TCA cycle. Hedgehog signaling through leptin represents a significant pro-fibrotic signal that may reprogram glucose metabolism, primarily converging on the transcription factor HIF-1α. Since AGEs result from hyperglycemia, TGF-β signaling enhances HSC expression of RAGE, rendering activated HSCs more susceptible to global glucose metabolism. B, The lipid metabolism of activated HSCs is characterized by the loss of retinyl ester-containing cytoplasmic droplets. HSCs utilize enhanced fatty acid β-oxidation to degrade the contents of lipid droplets within the autolysosome. Lipid droplets are transported to the autolysosome, where they undergo degradation. The contents of lipid droplets are degraded in the autolysosome under LAL activity, and REH activity is also increased in the lysosome, releasing free retinol into the extracellular space. SREBP-1c and PPAR-γ are adipogenic markers of quiescent HSCs that are downregulated upon activation. The conversion of some intracellular retinol to retinoic acid facilitates elevated transcription of RARβ and RXRβ. C, Free cholesterol in activated HSCs is partially absorbed through PCSK9, which interacts with low-density LRP5 and LDLR, promoting the degradation of endosomes and lysosomes. Free cholesterol enhances the expression of TLR4 and is converted into cholesterol esters through ACAT1, sensitizing HSCs to TGF-β signaling. oxLDL increases the expression of profibrogenic genes such as TNF-α and IL-1β through the pJNK. D, Metabolic stress responses in HSCs lead to the conversion of latent TGF-β into activated TGF-β. Unfolded proteins and TGF-β signaling trigger ER stress responses via IRE1α, resulting in increased expression of fibrogenic genes through ASK1/JNK signaling and the canonical splicing of XBP1 into its active form. This process promotes the expression of C/EBPβ, COL1A1, TGF-β, and XBP1 in the cell nucleus, leading to increased collagen accumulation. TGF-β signaling activates NADPH oxidase in a self-replicating cycle, producing H2O2, which promotes fibrogenic gene expression and activates latent TGF-β in extracellular spaces. Reduced nuclear receptor signaling, such as that of LXR, FXR, or THRα, is a hallmark of HSC activation. Nevertheless, active HSCs also exhibit increased PPARβ/δ, which stimulates HSC proliferation. ACAT1, acyl-CoA:cholesterol acyltransferase 1; ADAM17, ADAM metallopeptidase domain 17; AGE, advanced glycation end product; ASCT2, alanine serine cysteine transporter 2; ASK1, apoptosis-signal-regulating kinase 1; C/EBPβ, CCAAT-enhancer-binding protein; COL1A1, collagen type 1 alpha 1; DNL, de novo lipogenesis; FXR, farnesoid X receptor; GLUT, glucose transporter protein 1; HIF-1α, hypoxia-inducible factor 1-α; H_2_O_2_, hydrogen peroxide; HSCs, hepatic stellate cells; IL, interleukin; IRE1α, inositol-requiring enzyme 1 alpha; LD, lipid droplet; LDL, low-density lipoprotein; LDLR, low-density lipoprotein receptor; LRP5, low-density lipoprotein-related protein 5; LXR, liver X receptor; MCT4, monocarboxylate transporter 4; NOX, NADPH oxidase; oxLDL, oxidized low-density lipoprotein; PCSK9, proprotein convertase subtilisin/kexin 9; PKM2, pyruvate kinase M2; PPAR, peroxisome proliferator-activated receptor; RAGE, receptor for advanced glycation end product; RARα/β, retinoic acid receptor α/β; REH, retinyl ester hydrolase; ROS, reactive oxygen species; SREBP-1c, sterol regulatory element-binding protein-1c; TGF, transforming growth factor; THR, thyroid hormone receptors; TLR4, toll-like receptor 4; TNF, tumor necrosis factor; TREM2, triggering receptor expressed on myeloid cells 2; XBP1s, IRE1α-X-box binding protein 1.

HSCs are the primary fibrogenic cell population in the liver. In their quiescent state, HSCs function as the liver's retinol-storing pericytes and regulate sinusoidal blood flow during homeostasis. However, in response to liver injury, various damage-associated, inflammatory, and metabolic signals trigger the loss of retinol droplets, leading to transdifferentiation into a phenotype resembling that of myofibroblasts and subsequent activation of HSCs. This activated phenotype is characterized by increased migration, contractility, proliferation, and inflammatory signaling, as well as heightened production of extracellular matrix components. Ultimately, these changes result in abnormal extracellular matrix deposition, contributing to the development of fibrosis and cirrhosis. Significant metabolic reprogramming is necessary to facilitate the energy-intensive phenotypic shift toward a myofibroblast-like state, characterized by enhanced glycolysis, mobilization of lipid droplets, cholesterol metabolism, and activation of stress response pathways (**Figure 5**).

### Glucose metabolism

The activation of HSCs primarily depends on the reprogramming of their glucose metabolism. Notably, alterations in glucose metabolism not only define the myofibroblast phenotype but also contribute to HSC activation 164.

In comparison to their quiescent counterparts, activated HSCs exhibit higher rates of glucose utilization, enhanced glucose transport capabilities, and increased glycolytic activity in response to elevated extracellular glucose levels or purinergic signaling 165. This phenomenon is attributed to the upregulation of mRNA expression of glucose transporters, including glucose transporter protein 1 (GLUT1), GLUT2, and GLUT4 166. Notably, liver cancer cells, which primarily generate ATP through anaerobic metabolism, also demonstrate significant overexpression of GLUT1. GLUT1 represents a rate-limiting step in ATP synthesis 167. Additionally, activated HSCs show increased mRNA expression of rate-limiting glycolytic enzymes, including hexokinase-2 (HK2), pyruvate kinase M2 (PKM2), and fructose-2,6-bisphosphatase-3 (PFKFB3) 168. For example, the ablation of HK2 in HSCs completely inhibits CCl_4_-induced liver fibrosis in mice 169. The activation of HSCs and the formation of fibrosis may be mitigated by blocking the nuclear translocation of PKM2, which is essential for metabolic transition in HSCs and resembles the metabolic reprogramming processes observed in liver macrophages. Furthermore, exosomes produced by activated HSCs contain GLUT1 and PKM2 proteins 170. Hypoxia-inducible factor 1-α (HIF-1α) signaling enhances the production of exosomes by activated HSCs, particularly under hypoxic and inflammatory conditions 171.

The increased glycolysis observed in HSCs during cultivation is accompanied by the depletion of central carbon metabolites from the citric acid cycle. The activation of HSCs is characterized by high expression levels of pyruvate dehydrogenase kinase 3 (PDK3), which inhibits the conversion of pyruvate to acetyl coenzyme A (acetyl-CoA), thereby directing pyruvate toward lactate synthesis. Lactate plays a crucial role in HSC activation and the maintenance of myofibroblast phenotypes. Despite the upregulation of the lactate export pump, monocarboxylate transporter 4 (MCT4), the concentrations of lactate within activated HSCs remain elevated. Notably, inhibiting the intracellular accumulation of lactate reduces cell proliferation, decreases the expression of genes associated with the myofibroblast signature, and promotes lipid accumulation and lipogenic transcription 172.

Signaling pathways involving the interaction of hedgehog and leptin receptors play a crucial role in regulating glucose metabolism in HSCs 173. Hedgehog signaling is increasingly recognized for its involvement in tissue repair following injury, with hepatocytes producing hedgehog ligands in response to hepatic damage. These ligands activate nearby HSCs through the expression of the HIF-1α gene, which modulates energy metabolism by activating specific genes, including those encoding glucose transporters and glycolytic enzymes 174. Hypoxia triggers oxygen-independent ATP production processes, resulting in HIF-1α-mediated regulation of glucose metabolism, similar to the Warburg effect observed in activated HSCs 175. Furthermore, activated HSCs enhance the activity of glutaminase, the rate-limiting enzyme responsible for the hydrolytic deamination of glutamine to glutamate, through hedgehog signaling. Recent findings suggest that targeting the amino acid transporter alanine-serine-cysteine transporter 2 (ASCT2) may significantly impact glutaminolysis in HSCs. In humans with the HSC line LX-2, pharmacological depletion of ASCT2 resulted in decreased levels of glutamate and α-ketoglutarate, which subsequently limited the rate of oxygen intake and HSC activation 176.

HSCs are also activated by advanced glycation end products (AGEs), which are formed through non-enzymatic interactions between proteins and sugars 177. The stimulation of murine HSCs in culture by TGF-β leads to the development of the receptor for advanced glycation end products (RAGE), an effect that may be mitigated by inhibiting the mitogen-activated protein kinase (MAPK)/extracellular signal-regulated kinase (ERK) pathway 178. Cultured human HSCs exhibit increased expression of fibrogenic genes and produce higher levels of ROS when grown in a medium containing AGEs 179. *In vivo* studies demonstrate that rats receiving intraperitoneal AGE injections develop more severe liver fibrosis following bile duct ligation compared to those not receiving AGE injections. Although AGE treatment is not directly associated with fibrosis, it does elevate the levels of the protein α-SMA. This research suggests that AGEs and hyperglycemia alone may not be sufficient to induce fibrosis. However, they could exacerbate fibrogenesis triggered by other factors in the presence of tissue damage 180.

Changes in the glucose metabolism of HSCs are also influenced by epigenetic regulation 181. The histone methyltransferase G9a and DNA methyltransferase 1 (DNMT1) induce epigenetic modifications that shift HSC metabolism toward increased glycolysis. The profibrotic cytokine TGF-β stimulates the recruitment of these enzymes to chromatin in human HSCs 182. Furthermore, *in vitro* models of hypoxia-driven and TGF-β1-driven activation demonstrate that the simultaneous inhibition of DNMT1 and G9a restores glycolytic rates to those observed in quiescent HSCs 183.

### Lipid metabolism

Quiescent HSCs are characterized by the presence of vitamin A-rich lipid droplets, and the maintenance of this quiescent state requires both vitamin A metabolism and insulin signaling. Shortly after activation, HSCs release their lipid droplets, and over time, their lipid metabolism undergoes significant changes 184. The elimination of lipid droplets through autophagy appears to be essential for HSC activation, and this metabolically demanding cellular response may depend on this process (**Figure 5**) 185. Intracellular retinyl ester hydrolases (REHs) exhibit increased activity upon HSC activation, which subsequently triggers the release of retinol without a corresponding increase in the release of retinyl esters (REs). The absence of extracellular REH activity in activated HSCs supports the hypothesis that intracellular hydrolysis is responsible for retinol depletion 186. Bile salt-independent REHs, including patatin-like phospholipase domain-containing protein 3, adipose triglyceride lipase, and lysosomal acid lipase (LAL), are recognized as enzymatic mediators of RE release from lipid droplets 187. Notably, hormone-sensitive lipase, a key REH in liver adipocytes, is not significantly produced by HSCs and has minimal impact on lipid mobilization in these cells 188. Activated HSCs demonstrate increased hydrolase activity alongside a diminished capacity for retinol esterification, as HSC activation leads to a rapid decrease in lecithin-retinol acyltransferase expression. Retinol is thought to play a crucial role in maintaining the quiescent phenotype of HSCs through its interactions with the nuclear receptors retinoic acid receptor (RARβ) and retinoid X receptor α (RXRα). This phenomenon is primarily mediated by sterol regulatory element-binding protein-1c (SREBP-1c) and PPARγ, two adipogenic transcription factors that reduce their expression 189.

De novo lipogenesis (DNL) suppression, achieved through the inhibition of 1-aminocyclopropane-1-carboxylate (ACC) or fatty acid synthetase (FASN), is currently being investigated as a potential treatment for steatotic liver diseases. Studies in murine models have demonstrated that this approach can reduce hepatic fibrosis 190. Notably, multiple mechanisms contribute to these effects beyond the enhancement of hepatocyte metabolism. HSCs also exhibit increased expression of lipogenic pathway activity 191. When DNL is inhibited via ACC1/2 or FASN suppression, fibrogenic gene expression is significantly diminished *in vitro* in primary HSCs derived from both rats and humans 192, 193. This reduction is associated with decreased oxidative phosphorylation and glycolytic flow. However, the role of lipogenic pathways in non-metabolic hepatic fibrosis has not yet been established. Stearoyl-CoA desaturase-1 (SCD1) is the rate-limiting enzyme that converts saturated fatty acids into monounsaturated fatty acids and serves as a key regulator of fatty acid metabolic pathways 190. In both human and animal models of hepatic fibrosis, activated HSCs express SCD1, which promotes both liver fibrosis and liver cancer. Furthermore, metabolism-induced HSC activation triggers the production of ADAM metallopeptidase domain 17 (ADAM17), which cleaves the cell surface TREM2 protein. This process activates receptors expressed on myeloid cells and encourages HSC activation by inhibiting efferocytosis in the presence of TNF-α and IL-1β 194.

### Cholesterol metabolism

Cholesterol contributes to the pathophysiology of fibrosis formation in MASH because the accumulation of free cholesterol may activate HSCs 195. Moreover, MASH can be more effectively induced in mice that are fed a cholesterol-rich diet 196. When free cholesterol accumulates, TLR4 is upregulated in HSCs, making the cells more susceptible to activation by TGF-β 197. The low-density lipoprotein receptor (LDLR) and miR-33a, a microRNA that regulates cholesterol metabolism, are both elevated upon HSC activation and influence the accumulation of free cholesterol in these cells. The endosomal-lysosomal degradation of TLR4 is impeded by signals mediated by LDLR and miR-33a. Consequently, this reduces the levels of bone morphogenetic protein and activin membrane-bound inhibitor, a pseudoreceptor for TGF-β, thereby increasing the susceptibility of HSCs to TGF-β 198.

The significance of acyl-CoA:cholesterol acyltransferase 1 (ACAT1), the enzyme responsible for catalyzing the conversion of free cholesterol into cholesterol esters, has been extensively studied 199. The *in vitro* effects of free cholesterol on HSCs are significantly exacerbated, and liver fibrosis induced by CCl_4_ and bile duct ligation in mice is intensified by HSC-specific deletion of ACAT1. This pathway is dependent on the overexpression of TLR4, as the detrimental effects of ACAT1 deletion are mitigated in mice with conditional TLR4 deletion 200. Additionally, due to its complex formation with proprotein convertase subtilisin/kexin 9 (PCSK9), LDL-related protein 5 (LRP5) partially mediates the uptake of low-density lipoprotein (LDL) cholesterol in HSCs 201. Further research is needed to ascertain whether this cholesterol uptake pathway plays a role in HSC activation.

HSCs are rapidly activated by oxidized low-density lipoprotein (oxLDL). The uptake of oxLDL by HSCs is facilitated by scavenger receptors, specifically CD36 and lectin-like oxidized LDL receptor-1 (LOX-1). This process enhances the expression of profibrogenic genes, such as TNF-α and IL-1β, through the phosphorylation of c-Jun N-terminal kinases (JNK). Canonical Wnt signaling, recognized as an HSC activator, promotes the upregulation of LOX-1 expression 202.

### Metabolic stress responses

Metabolic stress responses, which encompass elevated oxidative stress, activation of the unfolded protein response (UPR), autophagy, ferroptosis, senescence, and nuclear receptor signaling, are associated with the activation of HSCs.

#### Oxidative stress

Increased oxidative stress, a hallmark of activated HSCs, is crucial for collagen synthesis and fibrogenic activation 203. The MAPK/ERK pathway is activated by several traditional HSC activators, such as TGF-β and PDGF, to the production of NADPH oxidase (NOX) enzymes 204. Hydrogen peroxide (H_2_O_2_), produced by NOX enzymes, plays multiple roles in liver fibrogenesis. Additionally, H_2_O_2_ facilitates the binding of CCAAT-enhancer-binding protein (C/EBPβ) to its binding sites in the type I collagen and TGF-β1 promoter, promoting collagen generation and increasing levels of fibrogenic TGF-β1. These alterations in the redox state, characterized by elevated levels of ROS, are essential for the activation of latent TGF-β 205. Consequently, in mouse models of liver disease, suppression or deletion of NOX enzymes reduces the progression of fibrosis and mitigates fibrotic responses in HSCs 206. Furthermore, a recent study has demonstrated that ROS generation induced by excessive iron in HSCs in the MASH model promotes fibrogenic HSC activation. This effect can be reversed by N-acetylcysteine, an antioxidant therapy 207.

β-oxidation activity has been observed to be elevated during HSC activation. Liver HSCs significantly enhance the activity of carnitine palmitoyltransferase 1A (CPT1A), the rate-limiting enzyme in the β-oxidation of medium- and long-chain fatty acids, in both human and murine models of CCl_4_-induced and metabolism-driven liver fibrosis. The progression of fibrosis can be mitigated through genetic suppression, pharmacological inhibition of CPT1A, and HSC-specific knockdown of CPT1A in mice with choline-deficient models of liver fibrosis 36.

#### The UPR and ER stress

One of the initial steps in HSC activation and hepatic fibrosis is the generation of UPR and ER stress 208. However, the ER stress pathway and the UPR alone are insufficient to fully activate HSCs. Evidence for this is provided by the reduced fibrosis formation observed in HSCs that overexpress the chaperone glucose-regulated protein, indicating that these processes are necessary for adequate HSC activation 209. Furthermore, stimulation with TGF-β1 can directly induce the phosphorylation of inositol-requiring protein 1α (IRE1α), which promotes the activation of the TGF-β1 downstream effector transcription factor C/EBPβ in LX-2 cells through apoptosis-signal-regulating kinase 1 (ASK1) and the phosphorylation of JNK 210. In parallel, TGF-β-induced overexpression of transport and Golgi organization 1, which facilitates HSC secretion of type I collagen, is mediated via the IRE1α-X-box binding protein 1 (XBP1s) pathway 211.

#### Autophagy, ferroptosis, and senescence

Autophagy, regulated by IRE1α-activated XBP1s, is closely associated with the activation of the ER stress pathway in HSCs 212. Additionally, macrophages may promote HSC autophagy. For instance, LAMs produce prostaglandin E2 (PGE2), which triggers HSC autophagy through its receptor, the PGE2 receptor 4 (EP4) 34. Autophagy in HSCs mobilizes lipid droplets, correlating with a reduction in retinol storage in stellate cells and an increase in the rate of β-oxidation 213. These findings indicate that autophagy serves as an energy source for HSC activation. Consequently, inhibiting autophagy by suppressing the PGE2 receptor EP4 reduces liver fibrosis induced by methionine-choline deficiency. Moreover, HSC-specific genetic deletion of autophagy-related genes (ATG)5 or ATG7 contributes to the inhibition of fibrogenic activation and decreases fibrosis in murine models of liver fibrosis induced by CCl_4_ and thioacetamide. Furthermore, the loss of ATG7 negates the effects of IRE1α-XBP1 signaling, establishing autophagy as the primary facilitator of ER-stress-driven HSC activation 214.

Programmed cell death, which include apoptosis, pyroptosis, necroptosis, cuproptosis, ferroptosis, and PANoptosis, occurs in various cell types and has distinct effects on hepatic fibrosis 215. Activated apoptotic proteases, such as caspase-3 and caspase-7, lead to the formation of apoptotic bodies, which can activate HSCs either directly or indirectly by stimulating macrophages. Hepatocyte death through mechanisms such as necroptosis, pyroptosis, ferroptosis, cuproptosis, and PANoptosis results in the aggregation of macrophages, monocytes, and DCs, leading to the release of DAMPs and inflammatory factors that further exacerbate the inflammatory response. Ferroptosis occurs in hepatocytes and promotes the formation of fibrosis. However, in HSCs, it exerts an anti-fibrotic effect. Hepatic fibrosis in mice, induced by bile duct ligation or CCl_4_, can be alleviated *in vivo* by the HSC-specific loss of ELAV-like RNA binding protein 1, a transcript essential for HSC ferroptosis, as well as by treatment with the ferroptosis inducers sorafenib or the anti-malarial medication dihydroartemisinin. Interestingly, sorafenib-induced ferroptosis does not occur in hepatocytes or macrophages, suggesting that it is exclusive to HSCs. Chronic iron overload, however, continues to stimulate hepatic fibrogenesis while inducing ferroptosis in both HSCs and hepatocytes.

The senescence of HSCs, a form of cell-cycle arrest in which the cells remain metabolically active, has been observed during the development of hepatic fibrosis and is initially associated with HSC deactivation 216. However, through the senescence-associated secretory phenotype, which promotes inflammation and creates a pro-tumoral liver environment, HSC senescence appears to be a significant source of inflammatory and fibrogenic signals during the later stages of fibrosis. Senescent HSCs, identified by the expression of the urokinase plasminogen activator receptor (uPAR), are derived from active HSCs in both human and mouse models of MASLD-related fibrosis, according to advanced single-cell analyses. Although it may not be entirely specific to senescent HSCs, the regulation of senescence by uPAR-specific senolytic chimeric antigen receptor T cells has been demonstrated in mouse models of hepatic fibrosis 217. Furthermore, hepatic fibrosis is reduced in both biliary and diet-induced mouse models of advanced hepatic fibrosis when cell-type-specific induction of senescence in HSCs is achieved through the conditional deletion of yes-associated protein (YAP) 1 11. This finding is consistent with previous research indicating that medication-induced suppression of YAP reduces CCl_4_-induced hepatic fibrosis in mice. However, other studies have found that YAP suppression has an opposing effect in ischemia-reperfusion injury, exacerbating hepatic fibrosis and delaying liver regeneration 218. Therefore, before developing specific treatment strategies, it is essential to further elucidate the precise role of HSC senescence in the progression of hepatic fibrosis.

#### Nuclear receptors signaling

The expression levels of nuclear receptors in HSCs are relatively low, and their role in HSC activation remains unclear. While HSCs do not express PPARα and PPARγ, they do express PPARβ. The activation of HSCs leads to an increase in PPARβ expression, which, in turn, stimulates HSC proliferation in response to CCl_4_-induced hepatic fibrosis 219.

Liver X receptor (LXR) ligands inhibit HSC activation in *in vitro* studies, indicating that LXR-deficient mice are more susceptible to methionine and choline deficiency, as well as CCl_4_-induced hepatic dysfunction. The liver pathology observed in LXR mutant mice is not altered by bone marrow transplantation from wild-type donors, suggesting that HSCs are indeed the mediating factor for these effects. The absence of LXR modifies how HSCs process lipids and metabolize retinoid compounds, resulting in larger lipid droplets and an enhanced capacity to respond to retinoic acid. This ultimately drives HSCs toward a more activated state 220.

FXR is expressed at lower levels in HSC and hepatic myofibroblasts. Endogenous FXR ligands have been shown to reduce the fibrogenic response of HSCs, but this effect occurs only in the presence of a small heterodimer partner, known as small heterodimer partner (SHP) 221. A different study finds no effect on HSC activation when rat or human HSC models are treated with obeticholic acid, a synthetic BA and FXR agonist. FXR signaling is predominantly observed in quiescent HSCs and diminishes upon fibrogenic activation 222. The activation of HSCs is further exacerbated by whole-body FXR deletion 223. The activation of HSCs is exacerbated by whole-body FXR deletion 220. Despite this, obeticholic acid appears to have minimal impact on the activation process. Therefore, the role of this bile acid receptor in HSC activation must be elucidated through targeted manipulation of FXR activity in HSCs.

Thyroid hormone receptors (THR)-α and β are nuclear receptors primarily activated by thyroid hormones (THs) to mediate their cellular responses 224. While THRβ is the mechanism through which THs exert their positive metabolic effects in hepatocytes, THRα is the predominant isoform in human and murine HSCs. THRα functions by inhibiting TGF-β signaling and fibrogenic activation in HSCs 225.

## Therapeutic implications

### Targeting liver inflammation and fibrosis

HSC metabolism is closely linked to the fibrotic response and hepatic inflammation. Currently, no medications targeting inflammatory or fibrogenic pathways have received regulatory approval for the treatment of liver damage. Several drugs, including the dual CCR2/5 inhibitor cenicriviroc and the ASK1 inhibitor selonsertib, have failed to demonstrate a reduction in fibrosis during Phase III clinical trials. This lack of efficacy is due to the complex nature of the underlying disease processes, which involve intricate interactions between inflammation, fibrogenic HSC activation, and hepatocellular damage 226. Consequently, preventing liver injury is likely essential for therapeutic effectiveness, as targeting a single pathway downstream in the disease cascade may be insufficient. The significant effectiveness of substantial weight loss following bariatric surgery is evidenced by the fact that 56% of individuals achieve the primary objective of MASH resolution without worsening fibrosis at the one-year follow-up, compared to only 16% in the lifestyle intervention group 227. Given that the metabolic responses of distinct cell types to liver damage overlap, there is an opportunity to target multiple cell types simultaneously and upstream in the disease cascade.

The development of innovative therapeutic options must take into account the etiology of the underlying liver disease. Given that macrophages play a significant role in the fibrosis associated with MAFLD and MASH, which have emerged as the most prevalent liver pathologies lacking effective treatment options worldwide, the majority of strategies discussed here focus on myeloid cells. However, different cell types may preferentially target other fibrotic liver diseases. For example, addressing T cell and other lymphocyte deficiencies in chronic hepatitis B virus (HBV) infection may represent the optimal treatment strategy to stimulate viral clearance and subsequently repair liver damage 228. Nevertheless, targeting macrophages may also prove beneficial. In animal models of HBV infection, subsets of Kupffer cells may circumvent the tolerogenic potential of the liver environment by enhancing liver T cell immunity through the detection of IL-2 and the cross-presentation of hepatocellular antigens 229. While managing auto-aggressive effector T cell responses requires further refinement, current techniques aim to increase the pool and functionality of T_reg_. T cells are also a significant focus in the treatment of autoimmune hepatitis 230. Controlling the accumulation or activity of neutrophils presents a potential therapeutic avenue for MetALD, as neutrophil activity is a major characteristic that correlates with liver inflammation and drives the progression of liver injury 231.

The treatment of fibrosis regression is currently under investigation, with a focus on facilitating the conversion of activated HSCs to quiescent HSCs and promoting the degradation of the extracellular matrix. This process may be regulated by the interactions between neutrophils and macrophages 8. Neutrophils can induce macrophages to switch to a restorative phenotype, which promotes liver tissue regeneration. In a study involving the accumulation of pro-inflammatory monocyte, the use of CCL2 inhibitors in mice demonstrates a transition of liver macrophages toward a recovery phenotype, thereby accelerating fibrosis regression 232. In a modest study involving nine individuals with compensated hepatic cirrhosis, the adoptive transfer of *ex vivo* differentiated restorative macrophages improved hepatic fibrosis in animal models and marginally decreased the end-stage liver disease score 233. Although this strategy appears safe in a small pilot study involving patients, it remains unclear whether it will be clinically effective and sustainable in the long term for the macrophage phenotype 234. Mesenchymal stromal cells (MSCs) have been investigated for adoptive cell therapy due to their immunoregulatory properties. Clinical trials are currently being conducted on mice with liver disorders to determine whether MSCs derived from bone marrow and MSC-derived extracellular vesicles can reduce liver inflammation and fibrosis 235. Preclinical models demonstrate a reduction in hepatic fibrosis following the adoptive transfer of chimeric antigen receptor T cells that target senescent hepatocytes via the senescence-associated urokinase-type plasminogen activator receptor 217.

The primary objective of therapies targeting the fibrogenesis process is to reduce or inhibit the activation of HSCs. TGF-β is considered a key target due to its significant role in HSC activation. However, pan-TGF-β blockade can lead to adverse effects, such as autoimmunity, because of its numerous systemic activities. These negative outcomes have resulted in the termination of several clinical trials involving monoclonal antibodies against TGF-β1 236, 237. A more prudent approach may involve regulating TGF-β locally at the site of action. Integrins containing αV subunits can activate latent TGF-β in the extracellular matrix, and the removal of this component has been shown to protect animals from fibrosis in various organs 238, 239. Although there is currently limited information regarding the efficacy of αV integrin inhibitors in humans, several drugs, including abituzumab, a pan-αV-binding antibody, are under investigation 240.

### Targeting glucose and lipid metabolism

One of the hallmarks of HSC activation is the increased glycolysis and the activation of lipogenic pathways, which reflect alterations in HSC metabolism associated with liver damage. Therefore, targeting these pathways may represent a promising strategy for treating hepatic injury, and several medications are currently undergoing preclinical or clinical research. Inhibiting rate-limiting glycolytic enzymes, such as hexokinase isoforms, is relatively straightforward, and this approach has demonstrated anti-fibrotic efficacy in animal models. PF-06835919 acts as an inhibitor of a rate-limiting enzyme in fructose metabolism and is currently in clinical trials for the treatment of multiple system lipodystrophies 241-243. Emerging data suggest that PKM2, a key regulator that induces the Warburg effect and serves as a rate-limiting glycolytic enzyme, is a suitable therapeutic target. *In vitro* studies indicate that the allosteric PKM2 activator TEPP-46 positively impacts hepatic inflammation and fibrosis by stabilizing its tetrameric complexes and preventing nuclear translocation 244, 245. Additionally, several medications targeting lipogenic pathways are currently under clinical investigation. In a human phase IIa trial, therapeutic suppression of ACC1 effectively reduces hepatic steatosis, fibrogenic gene expression, and liver inflammation in mouse models of MASH 246. In this therapeutic context, blocking SCD1 is also being tested in patients with MASH, although it has resulted in a non-significant reduction in liver fat. However, no discernible effects on liver fibrosis or inflammation have been observed 247. Another intriguing strategy involves the stimulation of AMP-activated protein kinase, a cellular energy sensor that inhibits anabolic pathways such as lipogenesis and may also confer beneficial cardiac effects 248, 249.

### Targeting metabolic stress responses

Metabolic stress responses, including ER stress, the UPR, autophagy, ferroptosis, and nuclear receptor signaling, represent distinct therapeutic targets. Inhibiting IRE1α and its downstream mediator, caspase-2, has been shown to protect against liver steatosis and inflammation in animal models 250. However, transient and low-quality activation of the ER stress system serves as an adaptive response in overweight individuals, and complete hepatic blockade can exacerbate liver damage. This consideration must be addressed when developing pharmaceutical therapies. The unique roles of autophagy and ferroptosis in HSCs may limit their applicability as treatment targets and necessitate further investigation. Deletion of ATG7 increases insulin resistance and ER stress, while overexpression of ATG5 extends longevity. Additionally, pharmacological strategies that activate autophagy have demonstrated protective effects against liver fibrosis, indicating that autophagy exerts a beneficial influence 251.

Special attention must be given to adverse reactions when addressing metabolic pathways, as drug activity is not limited to specific organs. For instance, PPAR agonists can effectively promote the morphology of anti-inflammatory macrophages and inhibit HSC activation. However, their clinical application is currently restricted due to cardiovascular issues, osteopenia, edema, fluid retention, and other adverse effects 252. Emerging drug delivery strategies may enable cell-type-specific modulation of metabolic processes, potentially reducing undesirable off-target effects. For example, dendrimer-graphene nanostars have successfully delivered the non-thiazolidinedione PPARδ agonist GW1929 to macrophages in a mouse model of CCl_4_-induced liver damage, shifting macrophage morphologies toward an anti-inflammatory, alternative phenotype and significantly preventing fibrosis formation 253.

Nuclear receptor activation is widely regarded as the most advanced strategy for targeting HSC immunometabolism in liver fibrosis. Research indicates that the benefits of FXR agonists are primarily mediated through their effects on myeloid cells 254. Obeticholic acid, an FXR agonist, is approved as a second-line therapy for primary biliary cholangitis 255, and both it and other FXR agonists, such as vonafexor are being investigated as treatments for MASLD fibrosis 256. However, the research and development of obeticholic acid as an anti-fibrotic treatment for MASH has been halted, as the FDA did not grant fast-track approval based on the existing evidence of its effectiveness and safety. Some trials of vonafexor have reported increased mild to moderate pruritus and elevated LDL cholesterol levels, necessitating further exploration and larger trials to address the unmet medical need in MASH 257. PPAR agonists, particularly those targeting PPARβ/δ, are well recognized for their efficacy in inducing anti-inflammatory macrophage polarization 258. Lanifibranor, a pan-PPAR agonist, is in late-stage clinical development and has shown positive results in a Phase IIb trial 259. Similarly, LXR activation promotes anti-inflammatory macrophage activation while suppressing HSC activation 260. Additionally, the THRβ agonist resmetirom has recently been granted expedited approval by the FDA for the treatment of MASH fibrosis based on favorable findings from the registrational phase 3 MAESTRO-NASH study 261. THRα is the predominant THR isoform in HSCs and innate immune cells, such as macrophages. Consequently, resmetirom exhibits both anti-inflammatory and anti-fibrotic properties, primarily mediated by hepatic metabolic changes rather than direct immune and metabolic actions 262. Since resmetirom is now an approved drug for MASH, other THR beta agonists, such as sobetirome, eprotirome, and VK2809, will need to be evaluated to determine which drugs perform best in combination with resmetirom. Future real-world studies will also be necessary to assess the added benefits of ad-hoc combinations of resmetirom in patients with chronic liver diseases.

## Future perspectives

HSC activation has opened up remarkable opportunities for novel therapies in patients with chronic liver diseases. The transition of HSCs from a quiescent state to a perpetuated phenotype is primarily characterized by fibrogenesis, contractility, proliferation, altered matrix degradation, chemotaxis, and immunological and inflammatory signaling. Equally important, various cell types—including hepatocytes, macrophages, biliary epithelial cells, liver progenitor cells, LESCs, NK cells, NKT cells, platelets, B cells, MAIT cells, γδ T cells, neutrophils, mast cells, and innate lymphoid cells—interact with HSCs to influence the progression of chronic liver diseases. Moreover, chronic liver diseases impact the metabolic and functional states of HSCs, which are largely regulated by glucose, lipid, and cholesterol metabolism, oxidative stress, UPR activation, autophagy, ferroptosis, senescence, and nuclear receptor activity. Therefore, a comprehensive understanding of the metabolic processes accompanying these differentiation pathways will be essential for developing targeted therapies.

Currently, numerous drugs targeting liver inflammation and fibrosis, as well as glucose and lipid metabolism and metabolic stress responses in HSC activation, are being investigated for the treatment of chronic liver diseases. For example, FXR, THR-β, and PPAR agonists regulate glucose, lipid, and bile acid metabolism, which positively influence chronic liver conditions such as MAFLD and MASH. By integrating emerging and state-of-the-art approaches, including single-cell 263 and molecule-resolution genome-wide molecular characterization 264, along with spatial (multi) metabolomics techniques 265, we may overcome the limitations of current strategies. This integration offers a comprehensive and multidimensional understanding of HSC metabolic processes, intercellular communication networks, and their relationships with metabolic cell states and disruptions in the steady-state cellular niche. Furthermore, in recent years, our understanding of cellular interactions in chronic liver disease, as well as the consequences of therapeutic targeting, has significantly expanded and extensively reviewed elsewhere. However, how intracellular metabolic regulation, especially in HSCs, largely enters these circuits remains to be determined.

Overall, it is crucial to attain a comprehensive understanding of the mechanisms that govern intracellular metabolic regulation of HSC in order to enhance diagnosis and treatment.

## Abbreviations

ACAT1: acyl-CoA:cholesterol acyltransferase 1
ACC: 1-aminocyclopropane-1-carboxylate
acetyl-CoA: acetyl coenzyme A
ADAM17: ADAM metallopeptidase domain 17
ADP: adenosine diphosphate
AGE: advanced glycation end product
ASCT2: alanine serine cysteine transporter 2
ASK1: apoptosis-signal-regulating kinase 1
ATG5/7: autophagy-related 5/7
ATP: adenosine 5' triphosphate
BA: bile acid
BMDMs: bone marrow-derived monocytes
CCL: C-C motif ligand
CCl_4_: carbon tetrachloride
CCR: CC chemokine receptor
C/EBPβ: CCAAT-enhancer-binding protein
COL1A1: collagen type 1 alpha 1
CPT1A: carnitine palmitoyl transferase 1A
CTGF: connective tissue growth factor
CXCL: C-X-C motif ligand
CX3CR1: CX3C chemokine receptor 1
CXCR: C-X-C chemokine receptor
DAMPs: damage-associated molecular patterns
DCs: dendritic cells
DNL: de novo lipogenesis
DNMT: DNA methyltransferase
ECM: extracellular matrix
EGF: epidermal growth factor
emKCs: embryonic KCs
ERK: extracellular signal-regulated kinase
ET1: endothelin-1
FASL: Fas ligand
FASN: fatty acid synthetase
FFA: free fatty acids
FGF: fibroblast growth factor
FGFR1: fibroblast growth factor receptor 1
FXR: farnesoid X receptor
GATA: GATA-binding factor
GLUT: glucose transporter protein 1
HBV: hepatitis B virus
HGF: hepatocyte growth factor
HIF-1α: hypoxia-inducible factor 1-α
HK2: hexokinase-2
HMGB1: high-mobility group box 1
H_2_O_2_: hydrogen peroxide
HSCs: hepatic stellate cells
5-HT: 5-hydroxytryptamine
IFN: interferon
IGF1: insulin-like growth factor 1
IGFBP5: insulin-like growth factor-binding protein-5
IL: interleukin
ILCs: innate lymphoid cells
IRE1α: inositol-requiring enzyme 1 alpha
JNK: c-Jun N-terminal kinases
LAL: lysosomal acid lipase
LAMs: lipid-associated macrophages
LD: lipid droplet
LDL: low-density lipoprotein
LDLR: low-density lipoprotein receptor
LOX-1: lectin-like oxidized LDL receptor-1
LPS: lipopolysaccharide
LRP5: low-density lipoprotein-related protein 5
LSECs: liver sinusoidal endothelial cells
LXR: liver X receptor
MAIT: Mucosal-associated invariant T
MASH: metabolic dysfunction-associated steatohepatitis
MASLD: metabolic dysfunction-associated steatotic liver disease
MAPK: mitogen-activated protein kinases
MetALD: metabolic dysfunction-associated alcoholic liver disease
MCT4: monocarboxylate transporter 4
MCP1: monocyte chemoattractant protein 1
MDR2: multidrug resistance protein 2
MMP: Matrix metalloproteinase-2
MoMF: monocyte-derived macrophage
MSCs: mesenchymal stromal cells
NK: natural killer
NKG2D: NK receptor group 2 member D
NKT: natural killer T
NLRP3: NACHT: LRR and PYD domainscontaining protein 3
NOS: nitric oxide ynthase
NOX: NADPH oxidase
oxLDL: oxidized low-density lipoprotein
PAMP: pathogen-associated molecular pattern
PCSK9: proprotein convertase subtilisin/kexin 9
pDCs: plasmacytoid dendritic cells
PDGF: platelet-derived growth factor
PDGFRβ: platelet-derived growth factor receptor β
PDK3: pyruvate dehydrogenase kinase 3
PFKFB3: fructose-2,6-bi-sphosphatase-3
PGE2: prostaglandin E2
PKM2: pyruvate kinase M2
PPAR: peroxisome proliferator-activated receptor
RAGE: receptor for advanced glycation end product
RARα/β: retinoic acid receptor α/β
REs: retinyl esters
REH: retinyl ester hydrolase
ROS: reactive oxygen species
RXRα: retinoid X receptor α
SAM: scar-associated macrophage
SCD1: stearoyl-CoA desaturase-1
SHP: small heterodimer partner
α-SMA: α-smooth muscle actin
SREBP-1c: sterol regulatory element-binding protein-1c
TCA: Trichloroacetic acid
TCF21: transcription factor 21
TGF: transforming growth factor
TGF-βRII: TGF-β receptor II
TGR5: Takeda G protein-coupled receptor 5
T_H_: T helper
THR: thyroid hormone receptors
THs: thyroid hormones
TLR: toll-like receptor
TNF: tumor necrosis factor
T_reg_: regulatory T
TREM2: triggering receptor expressed on myeloid cells 2
TRAIL: tumour necrosis factor-related apoptosis-inducing ligand
uPAR: urokinase plasminogen activated receptor
UPR: unfolded protein response
UTC: unconventional T cell
VEGF: vascular endothelial growth factor
XBP1s: IRE1α-X-box binding protein 1
YAP: yes-associated protein
